# High-Throughput, Time-Resolved Mechanical Phenotyping of Prostate Cancer Cells

**DOI:** 10.1038/s41598-019-42008-0

**Published:** 2019-04-05

**Authors:** Yuri Belotti, Serenella Tolomeo, Michael J. Conneely, Tianjun Huang, Stephen J. McKenna, Ghulam Nabi, David McGloin

**Affiliations:** 1 University of Dundee, SUPA, School of Science and Engineering, Dundee, Scotland UK; 2 University of Dundee, School of Life Sciences, Dundee, Scotland UK; 3 University of Dundee, School of Medicine, Dundee, Scotland UK; 4 University of Technology Sydney, School of Electrical and Data Engineering, Sydney, Australia

## Abstract

Worldwide, prostate cancer sits only behind lung cancer as the most commonly diagnosed form of the disease in men. Even the best diagnostic standards lack precision, presenting issues with false positives and unneeded surgical intervention for patients. This lack of clear cut early diagnostic tools is a significant problem. We present a microfluidic platform, the Time-Resolved Hydrodynamic Stretcher (TR-HS), which allows the investigation of the dynamic mechanical response of thousands of cells per second to a non-destructive stress. The TR-HS integrates high-speed imaging and computer vision to automatically detect and track single cells suspended in a fluid and enables cell classification based on their mechanical properties. We demonstrate the discrimination of healthy and cancerous prostate cell lines based on the whole-cell, time-resolved mechanical response to a hydrodynamic load. Additionally, we implement a finite element method (FEM) model to characterise the forces responsible for the cell deformation in our device. Finally, we report the classification of the two different cell groups based on their time-resolved roundness using a decision tree classifier. This approach introduces a modality for high-throughput assessments of cellular suspensions and may represent a viable application for the development of innovative diagnostic devices.

## Introduction

Prostate cancer (PCa) is one of the most prevalent forms of male cancer throughout the world^1,2^, and is the fifth largest cause of cancer-related deaths in men. In part, due to higher life expectancy rates, these figures increase in countries such as the USA and those in Western Europe^3^. In the UK, for instance, PCa is the most widespread cancer in men^4^; its incidence rates have increased by 155% since the late 1970s, with 46,690 new cases registered in 2014 alone^5^. The current diagnostic tests lack sensitivity and specificity sufficient to distinguish between a benign enlarging gland and cancerous changes, typically leading to overdiagnosis^6^. To prevent just one death in the United States alone, it has been estimated that the cost of screening with prostate specific antigen (PSA) and lifetime treatment costs of identified prostate cancer is approximately US$5 million^7^. Diagnostic and therapeutic decisions are commonly driven by anomalous levels of PSA in patients’ blood while, on the other hand, this antigen is known to be prostate-specific but not cancer-specific^8^. The standard screening threshold of 4.1 ng/mL has recently been challenged and accurate cut off values indicative of obtaining a biopsy remain controversial^9^, since raised levels can be a consequence of an enlarged or inflamed prostate^3^. Hence, reliable biomarkers for the early-stage detection and characterisation of prostate cancer are not available, and this results in unnecessary and extremely invasive treatment. New methods are required to improve diagnostic and prognostic care pathways.

Various diagnostic techniques have been developed over the last decades, where biochemical markers were investigated to assess the presence and stage of the disease. However, biophysical properties could also represent a viable alternative. For instance, it has been shown that measuring cellular elasticity not only allows one to discriminate between cancerous and healthy cells, but also to determine their metastatic potential: more aggressive cells can, for example, have a different stiffness compared with less aggressive ones^8,10^. There is now significant evidence that the examination of the a cell’s response to external mechanical stress offers meaningful data about the cytoskeleton^11^. In turn, changes in the cytoskeleton can be considered to have resulted from disease^12–15^ and so are able to act as a label-free biomarker for cell-cycle stage^16^, differentiation state of stem cells^17^ and the metastatic state of cancer cells^10,12^.

Various techniques have been recently developed with the goal of investigating cellular mechanical properties^10,12,17–20^. Dudani *et al*.^21^ discriminated between Jurkat leukemia cells, MCF7 breast cancer cells, and HeLa cervical cancer cells based on their cellular deformability, using a microfluidic hydrodynamic stretcher. An earlier work by Faria *et al*.^8^ showed how prostate cells of different stages in the disease progression can be discriminated based on their elastic properties. Using atomic force microscopy (AFM) they could discriminate between primary benign prostate hyperplasia (BPH) and two well-characterised and widely used prostate cancer cell lines, LNCaP and PC-3. Notably, they were not able to find any mechanical difference between normal PNT2 and PC-3 cell lines which are known to have the highest metastatic potential^22^.

Here we describe a microfluidic device capable of characterising the time-resolved deformability of single cells subjected to hydrodynamic stress. We report the results of the mechanical characterisation and phenotyping of prostate cell lines: PNT2, commonly used as healthy control cells and DU145, known to possess a moderate metastatic potential^22^. High-speed imaging was used to monitor the highly dynamic interaction between single cells and a pinching flow. A contour tracking algorithm was developed to quantify cellular deformation over time as a consequence of the applied hydrodynamic load. Furthermore, we implemented a finite element method (FEM) computational model to characterise the spatio-temporal profile of the forces responsible for the cell deformation in our device. Finally, we classified the two cell groups as a result of their different temporal mechanical response using a decision tree classifier.

## Results

### Time-resolved single cell deformation

**Figure 1:**
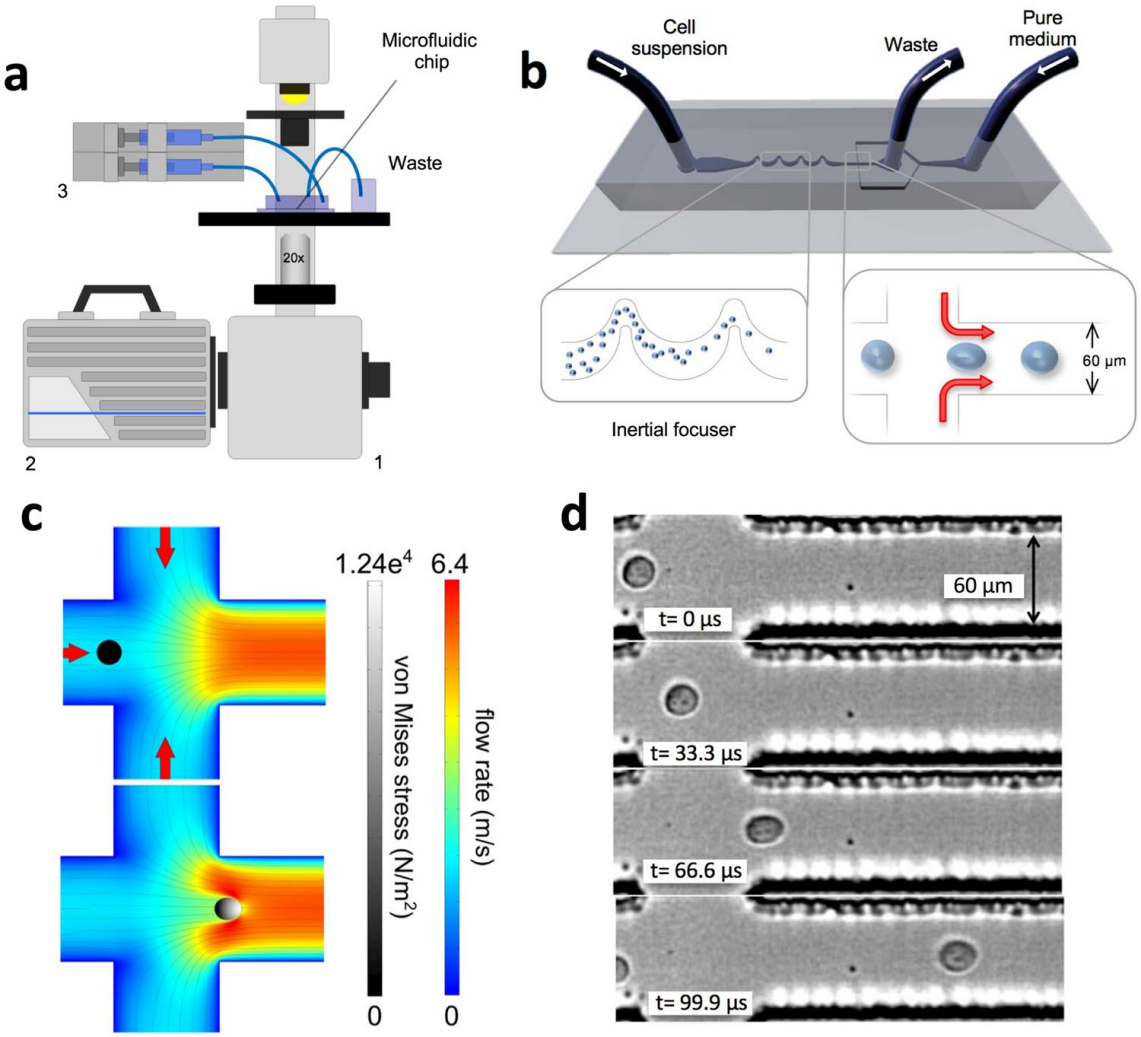
Experimental set-up and mechanical phenotyping. (**a**) Schematic diagram of the hydrodynamic stretching platform. The microfluidic chip is on a standard inverted microscope (1). A high-speed camera (2) is used to record the cell deformation and a syringe pump (3) is used to independently control the flow rate that transports the cell suspension and the pure medium used to create the pinching flow. (**b**) Schematic diagram of the microfluidic chip. The inertial focuser focuses the cell suspension down to a single streak of evenly spaced cells towards the centre of the channel, which are then deformed by the pinching flow. The output of the chip is simply routed and collected into a container. After the mechanical stretching, cells are still viable (survival rate of 94.7 ± 2.2%, averaged over three independent experiments) and therefore the output suspension could potentially be subjected to further analyses. (**c**) Simulated fluid velocity profile at the mid-plane of the channel at the junction where the pinching streams join the main channel, in the case of an unperturbed cell and a cell at the position of maximum deformation, respectively. The von Mises stress profile in the cell is represented by brightness (grey scale). (**d**) A DU145 cell deforms as a result of the pinching flow. Before entering the pinching region, the cell is unperturbed and hence its shape is almost spherical. In the second frame the cell starts to experience the effect of the pinched flow and it is slightly deformed. The deformation reaches its maximum in the third frame and it relaxes to a residual deformation.

To explore the cell mechanics we made use of our microfluidic platform (Fig. 1a,b), which exploits hydrodynamic effects to order and deform single cells at high throughput, based on extending, by modifying the device design, previous work by Dudani *et al*.^21^. In our device, the cell suspension and cell-free medium are loaded into the microfluidic chip (Fig. 1b) through two separate inlets. The cell suspension first flows through a filtering region that prevents large clusters or unwanted particulates from clogging the downstream microchannels. The flow rate transporting the cells is controlled by a digital syringe pump. Cells flow through an inertial focuser (as shown in Fig. 1b), which comprises a series of bends of different width and radii of curvature and^23^. This microfluidic element focuses the cell suspension to a single, evenly spaced, line of cells at the centre of the microchannel^24^. The main channel intersects two perpendicular and symmetric side arms that are connected to the second inlet from which pure culture medium is constantly loaded into the chip. The flow rate in these arms is controlled independently to that of the cell flow by a separate module of the syringe pump. As the side flows pinch the main flow, each cell becomes subjected to a space-dependent stress as it flows through the pinching region. Therefore, in the cell’s frame of reference, a time-dependent stress, dependent on position along the axis of the channel is observed. Figure S2 describes the shear stress and the net absolute pressure experienced by a cell as it travels across the various regions of the chip in proximity of the pinching region. To impart a measurable cellular deformation, flow velocities in the order of 6 m/s are required; therefore, a high-speed imaging system capable of recording at 300,000 frames per second (fps) was needed to accurately acquire these fast dynamics. High-speed image sequences were analysed using our automated cell tracking algorithm. The perimeter and the area of each cell are automatically extracted in each frame and the time-dependent *roundness* profiles can be computed and used as a *biomarker*. Importantly, using a fully coupled fluid-structure interaction simulation we found that the size of the cell greatly affects its interaction with the surrounding fluid; specifically, larger cells are subjected to greater stresses and hence exhibit greater deformations (Figs S3–S6). This is similar to what was previously shown by Otto *et al*.^25^. Simulations were run for cells sizes over a range covering what was typically seen in our experimental measurements (radii of 5–10 µm). In addition, three different shear moduli were chosen to represent “compliant”, “medium” and “stiff” cells which extended over the range of maximum deformations as seen in our experimental data. When normalised for initial radius size, each cell shear moduli group displayed a similar trend with changing radii. An exponential fit was made to these normalised values (Fig. S5b).

To test the feasibility of using this exponential function fit as a correction factor it was applied to time resolved roundness profiles (from FEM model solutions) of cells with various radii and a shear modulus of 45 kPa (Fig. S6).

The robustness of this correction was established by application to the peak deformation values obtained from simulations of cells with shear moduli of 40 kPa (compliant), 50 kPa (medium) and 60 kPa (stiff), and initial radii ranging from 5 µm to 10 µm (Fig. S7).

### Mechanical phenotyping of prostate cells via TR-HS

The control cell line PNT2 and the metastatic prostate cancer cell line DU145 were characterised using our microfluidic platform. Twelve independent experiments, over three consecutive passages (four technical repeats for each biological repeat) were performed. In total, 2108 DU145 and 2438 PNT2 cells were individually analysed.

**Figure 2:**
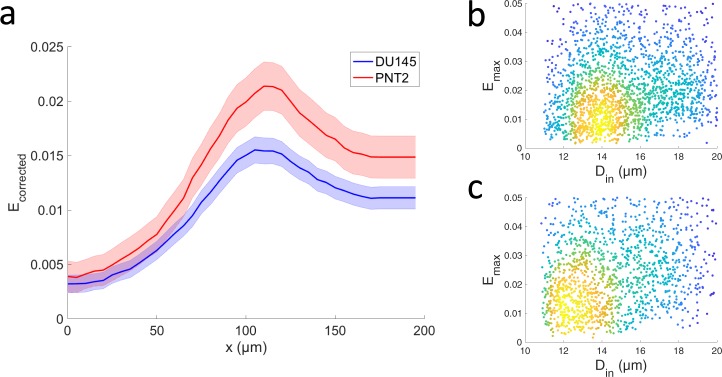
Mechanical characterisation of metastatic and healthy prostate cell lines. (**a**) Temporal profiles of the cellular mechanical response estimated as median deviation from the perfect roundness $\,{E}_{corrected}=1-{R}_{corrected}$ for DU145 (n = 2199) and PNT2 (n = 2852) cells. The error bars represent the 95% confidence intervals. Data collected over 12 independent experiments. The two profiles were significantly different at all positions (p < 0.0001, Mann-Whitney U test). (**b**,**c**) Density scatterplots of the *E*_*corrected*_ (maximum deviation from perfect roundness) versus the initial diameter *D*_*in*_ of each of the DU145 and PNT2 cells analysed in (**a**). The density of the data points in (**b**,**c**) is given by colour, with blue corresponding to the lowest density and yellow to the highest.

In Fig. 2a two distinct spatial profiles determined via TR-HS are shown. They represent the evolution of the median ${E}_{corrected}=1-{R}_{corrected}$ (*i*.*e*. the deviation from perfect roundness) along the microchannel, for each cell group. The size-corrected roundness, defined as ${R}_{corrected}=1-(1-R){e}^{-0.138r}$ (Fig. S6), where R is the cell roundness and r is the cell radius, became necessary as a statistically significant difference in cell diameter between the two groups was observed, as shown in Fig. 2b,c. In fact, the diameter of the unperturbed cells of DU145 (15.1 ± 0.1, mean ± s.e.m.) was significantly different (p < 0.0001, Z = −7.38, Mann-Whitney U test) from the diameter of PNT2 (15.6 ± 0.1, mean ± s.e.m.). To estimate the differences between the *E*_*corrected*_ spatial profiles we performed a Mann-Whitney U test at three representative position: initial, maximum and final, corresponding to the spatial position 5 µm, 150 µm, 250 µm along the microchannel. The *E*_*corrected*_ was highly significantly different at all positions (p < 0.0001, Z = −6.726, −11.398, −10.872, respectively). The control group PNT2 exhibited higher deformability, corresponding to a softer phenotype. Moreover, the profiles exhibit a residual deformation after the maximum *E*_*corrected*_ peak. This is indicative of a viscoelastic response to the applied stress, information which could potentially allow identification of a specific cell type or sub-populations. Figure 2b,c show the maximum deviation from perfect roundness ${E}_{max}=1-{R}_{min}$, where *R*_*min*_ is the minimum of the roundness profile for each cell. The *E*_*max*_ of DU145 (0.03 ± 0.0008, mean ± standard error in the mean (s.e.m)) was significantly different (p < 0.0001, Z = −15.3, Mann-Whitney U test) from that of PNT2 (0.06 ± 0.0015, mean ± s.e.m.).

Finally, we tested whether the mechanical properties of these cell lines were affected by the number of cell passages during culture and no statistically significant effect was found (*p* > 0.05, Z = −18.6, Mann-Whitney U test).

**Figure 3:**
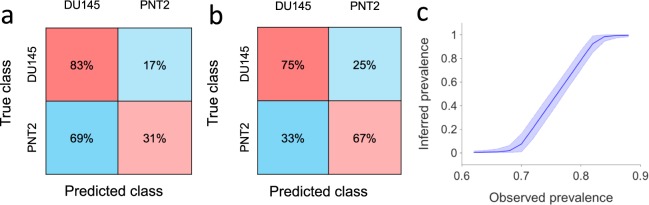
Cellular classification. Classification test results obtained by decision tree classification using as features the size-corrected roundness at the initial position, the final position, and at the point of the maximum deformation. (**a**) Confusion matrix for classification of individual cells (AUC = 0.58). (**b**) Confusion matrix for classification of groups of five consecutive cells (from the same class) based on features obtained from their average corrected roundness profile (AUC = 0.72). (**c**) Cell line prevalence in a mixed sample (*n* = 5000) obtained using Bayesian inference based on the specificity and sensitivity reported in (**a**). Plotted are posterior densities (means and 95% posterior probability intervals) for the inferred prevalence of ‘positive’ cells. Densities were computed for observed prevalence values that corresponded to the cell classifier classifying *k* cells as positive where *k* ∈ {3100, 3200, …, 4300, 4400}.

Using a decision tree classification we were able to classify DU145 and PNT2 cell lines by considering their temporal corrected roundness profiles. Due to the noisy outcome of the automatic segmentation and tracking (as shown for five cells in Fig. S9), we decided to obtain smoother temporal profiles by averaging the roundness profiles of each group of five consecutive cells passing through the pinching flow. Using the values of the corrected roundness averaged over five consecutive cells, we were able to successfully classify the two cell groups. Specifically, taking values of their corrected roundness profiles at the position of initial, maximum and final deformation resulting classification, we obtained a classification accuracy of 71.4%, with a specificity of 67% and a sensitivity (or True Positive Rate, TPR) of 75%, respectively (Fig. 3b). The use of these three representative values instead of the entire temporal profiles reduced the time of the classification process consistently without affecting its accuracy. Classification of individual cells based on their noisy roundness features resulted in a lower accuracy of 61.5% (83% sensitivity and 31% specificity) (Fig. 3a).

Furthermore, we sought to understand whether our single cell classifier could be used to infer the relative prevalence of a cell line in a sample containing a mixture of the two cell lines. For illustrative purposes, consider using it to analyse a mixed sample of two populations in which the cells are mixed in 50:50 proportion. Specifically, consider a sample of 5000 cells of which 2500 are ‘positive’ and 2500 are ‘negative’. The cell classifier’s low specificity means that it will certainly classify the majority of cells as positive. The number of positive classifications that would be obtained in such a setting follows a binomial distribution and has mean 3800 with 95% posterior probability interval (3740, 3860). Conversely, given an experiment in which the cell classifier was applied to a mixed cell sample resulting in a certain number of positive cell classifications, we could use Bayesian inference to infer the true prevalence of positive cells in the sample (see Methods). For example, if 3800 cells were classified as positive by the classifier, a run of the inference algorithm gives an inferred prevalence of 49.9% with 95% posterior interval (41.4%, 58.4%). Figure 3c shows prevalence distributions inferred in this manner across the entire range of feasible observed prevalence. It suggests that even a weak cell classifier, such as the one considered here, might be used to infer prevalence in large mixed samples.

### FEM modelling of the cell-fluid interaction

To understand how cells and the surrounding stream are mutually influenced during the cellular deformation, we implemented a fully coupled, time dependent fluid-structure interaction finite element model (FEM) of the TR-HS (see Methods).

Figure S2 shows the time evolution of shear stress and net pressure acting on a cell passing through the pinched flow region in the TR-HS. The flow rate around the cell and the von Mises stress in the cell are also depicted in Fig. S2. The simulation also allowed us to estimate the relationship between the cell size, cell speed and the hydrodynamic forces, as shown in Figs S3–S5.

Based on the magnitudes of the cell deformation observed during the experiments, our model suggests a cellular shear modulus (*G*) in the order of 50 kPa. If we consider cells to be isotropic, and assuming the Poisson ratio (*ν*) being 0.5^12^, we can then estimate that the elastic modulus (E = 2G(1 + v)), would be in the range of 150 kPa. On the other hand, by performing AFM force spectroscopy on a sample of DU145 cells (N = 72), Young’s modulus of 799 ± 545 Pa (mean ± s.d.) was recorded. The obtained Young’s moduli were corrected with a double-contact model which accounts for compression due to contact between the bottom of the cell and the hard substrate of the measuring dish^26^.

## Discussion

We developed a microfluidic platform for investigating the dynamic mechanical response of cells in suspension. The working principle is based on hydrodynamic stretching introduced by Dudani *et al*.^21^, where cell deformation is imparted by a pinching flow. In our implementation of the system an additional inlet has been added to allow for an independent control over the flow rates of the pinching flow with respect to the flow rate of the cell solution. This configuration, despite the additional inlet, allowed us to eliminate the region of the chip where the cell-free fluid is siphoned off from the main flow described by Dudani *et al*.^21^. This helps to reduce clogging in this area of the system and therefore increases the reliability of the single measurement as well as improving the usability of a device over repeated measurements. In fact, even a partial obstruction of the channels could lead to big changes in the hydrodynamic forces acting on the cells, and therefore affect the reproducibility of the outputs. Therefore, the absence of obstructions needs to be guaranteed. Furthermore, the additional inlet allows for a fine-tuning of the forces acting on the cells, by changing the flow rate ratio between the main and side arm flows. In addition, different fluids can potentially be loaded through each inlet. The use of our Time-Resolved Hydrodynamic Stretcher (TR-HS) enabled us to discriminate two different prostatic cell lines, DU145 (cancerous) and PNT2 (healthy), based on their time-resolved roundness profiles.

Various studies have shown that cancer cells are softer than healthy cells and that the negative correlation can be found between cell stiffness and aggressiveness^8,10^. It is believed that the decreased stiffness confers aggressive cancer cells the ability to more easily invade neighbouring tissues^27^. Based on this hypothesis it was expected that the moderate metastatic potential DU145 cells would be softer than the PNT2 cells. However, our results show the opposite. One possible reason for this is that these cells lines have different origins (PNT2 cells are derived are normal adult prostatic epithelial cells, while the DU145 are derived from brain metastatic cells) and hence it is not possible to draw any mechanistic connection between their mechanical phenotype and physiology. Another explanation would be that our technique measures the stiffness at very short time-scale (µs). In this case it is not possible to compare our results with those of techniques such as AFM which allows for assessing cellular mechanical properties dominated by steady-state viscosity.

In addition to previous methods^13,21,25^, where a static mechanical measurement is commonly used to generate flow cytometry-like density scatterplots, to characterise mechanical fingerprints of individual cellular group, we introduce an alternative measurement modality that is based on spatial profiles where the time-dependent mechanical response of large number of cells can be assessed and used as a biomarker for discriminating among different cellular groups. A roundness profile represents the mechanical response of the cell to the hydrodynamic load experienced, at each position, while travelling along the channel. The TR-HS bridges the gap between microfluidic-based single-cell assays, and measurements previously demonstrated only through optical-based technologies, such as the optical stretcher^28–31^, by which changes in cellular viscoelasticity of whole cells in suspension could be assessed. Our method has the capability of the latter technology of capturing time-resolved mechanical information, combined with the high-throughput of the former.

Additionally, we sought to characterise how cells interact with the surrounding flow. We implemented a fully coupled, time dependent, fluid-structure interaction finite element model (FEM) of the TR-HS. As cells never reach a steady-state deformation in this channel configuration, this enabled us to better describe the two-way interaction between the pinching flow and a deforming cell, which would prove challenging using analytical methods. Furthermore, this simulation allowed us to investigate the effect of the cell size and relative elasticity on the resulting measurement of cell roundness. Hence, a corrective factor for the cell roundness in this TR-HS geometry was determined to account for the size-dependent cell deformability.

The model also allowed us to investigate the scale of the shear stress acting on a cell flowing through the pinching flow. The estimated cellular elasticity appears to contrast our AFM spectroscopy data, ~150 kPa and ~370 Pa respectively. A similar observation was made by Mietke *et al*.^32^ in their microfluidic based real-time deformability cytometry study, where it was suggested that such a discrepancy may be explained by the frequency dependence of cellular elasticity. Evidence has been published in the literature which suggests that the elastic response of cells follows a weak power law in time, typically with exponents of 0.1 to 0.5 (Kollmannsberger *et al*.^33^). Using this relation and a timescale of the order of ~ms, Meike *et al*.^32^ drew some validity to their high estimated elasticity values, where they noted AFM derived values of the order of 0.17 kPa compared to ~1.5 kPa from their RT-DC experiments on HL60 cells. Following a similar reasoning, we can use the following relation to extrapolate from our AFM measured values of ~370 Pa,

$${K}_{i}={K}_{0}{({t}_{0}/{t}_{i})}^{\alpha }$$

where, K_i_ is the expected modulus, K_0_ is the experimentally measured modulus (i.e. AFM), t_0_ and t_i_ are the respective strain rates (i.e. ~1 s^−1^ for AFM and ~1 *μ*s^−1^ for TR-HS), and *α* is the power law exponent. Figure S8 shows a plot of this power law relation where, considering the short timescales involved in our hydrodynamic cell stretching, estimated elastic moduli in the range 1–300 kPa may be expected depending on the exponent chosen; this fits well with the FEM simulation results.

It has been suggested that materials, such as biological cells, which exhibit power-law like responses may be more accurately described by a distribution of characteristic relaxation times and corresponding shear moduli^34,35^. Additionally, it has recently been proposed that an incomplete deformation recovery, such as that seen in our experimental deformation profiles (Fig. 2a), is due to an additive plastic deformation process that follows a similar power-law response^36^. In the future, a more complete treatment of the physical properties of the cells in our FEM model would include appropriate treatments to account for these more complex behaviours.

One important aspect to consider is that our AFM measurements were done in PBS while the microfluidic measurements were done in culture medium. This might contribute to a different mechanical behaviour as culture medium contains growth factors which are known to activate Rho/Rac pathways^37,38^.

Biological samples have an extremely heterogeneous nature and cellular physiological properties change with time and experimental conditions. Therefore, a key requirement for a robust diagnostic tool is the capability to acquire measurements from large populations, in a short period of time, at constant environmental conditions, without excessive sample handling.

A disease might involve a small sub-population of cells, and therefore the capability of detecting subtle cellular anomalies is a key requirement. Our method represents a novel solution to these diagnostic requirements (also demonstrated in other diseases using similar technologies^13,21,25^). Only small volumes of cell solutions are required, and several thousands of cells can be analysed in a few seconds. Additionally, no labelling or molecular probes are required, reducing reagent batch variability as well as preparation time, labour and costs^21^.

Utilising machine learning, classification of samples of five cells into the two cellular populations based solely on mechanophenotypic information with a 71.4% accuracy (75% sensitivity and 67% specificity) was achieved. This value, which is reasonably high for such a small difference in roundness between the two cell lines, could be improved substantially in the case of greater differences in mechanical properties between different sample groups. Tests will be carried out with prostate primary cells to understand whether differences in stiffness can be used as a biomarker of the disease. The single-cell roundness profiles produced by the automatic segmentation and tracking were noisy; classification accuracy could be improved by reducing the noise associated with the cell segmentation through a further refined algorithm. However, even a weak cell classifier could be used to infer the relative prevalence of a cell line in a sample containing a mixture of two cell lines. Figure 3c shows this for an illustrative case in which 5000 cells are classified using a classifier with the specificity and sensitivity reported in Fig. 3a. Shown are the mean posterior probability (with 95% posterior probability intervals) of the true prevalence of a cell line given the observed prevalence, i.e. given the proportion of the cells classified as belonging to that cell line by the cell classifier. These posterior densities were computed using Bayesian inference (see Methods).

The aggressiveness of a prostate tumour is currently assessed using the Gleason scoring system that evaluates the morphological architecture of histologic samples^39^. The arrangement of the cells within the prostatic tissue sections is visually inspected and a score is assigned based on their pattern. However, this method is characterised by a high degree of subjectivity, as well as intra-observer and inter-observer variability^39,40^. Moreover, because of the heterogeneous and multifocal nature of this disease, the likelihood of missing the lesion during the biopsy is high, with a high morbidity associated with invasive biopsy procedure^41^. Using our method, cells shed from the prostate into urine could be analysed instead; this would allow for an additional non-invasive guidance for treatment decisions, enabling a personalised medicine approach^13^. Although high-speed imaging is still an expensive technology, its increasing adoption in many areas of science and technology should lead to the release of faster, as well as cheaper devices in the near future. Our high-speed camera allows the user to store a video containing several hundred thousand frames, that can be transferred to a PC for further analysis with a delay of a few minutes. However, our image analysis algorithm requires a few hours to analyse the content of the images. These delays prevent our method from direct adoption into medical settings. On the other hand, the combination of real-time acquisition methods (similar to that shown by Otto *et al*.^25^) and quicker image segmentation algorithms, will help to improve our technique substantially.

Cell mechanics is an important alternative method to quantify differences among cell types. The label-free biomarker reported in this work could be exploited to develop screening devices capable of achieving, alone or in combination with existing screening modalities, better early-stage diagnostics based on the analysis of bodily fluids.

## Methods

### Device fabrication

Standard photolithographic techniques were employed to develop the moulds of the microfluidic chip. Replicas were obtained by soft lithography using polydimethylsiloxane (PDMS) (Sylgard 184 Silicone Elastomer Kit; Dow Corning Corporation). A 1 mm biopsy puncher (Harris Uni-Core) was used to open ports punched through the inlet and outlet. The complete microfluidic devices were sealed using a glow discharge system (Agar Scientific) where the micropatterned PDMS layers were bonded to standard microscope glass slides (VWR) and successively annealed at a temperature of 80 °C for 1 hour.

### Hydrodynamic manipulation

Cell suspensions are introduced into the device through the *cell loading inlet* (Figs 1b and S1), and the pinching flow is loaded through a second inlet (Figs 1b and S1). The two fluxes are independently controlled using a syringe pump (neMESYS, Cetoni) and introduced into the chip via 1/16 inches PTFE tubing (Sigma-Aldrich). The cell solution moves through a filtering region (Figs 1b and S1) which helps to avoid unwanted objects obstructing the channels downstream of it. An inertial focuser is located downstream of the filtering region. This component comprises alternating bends with different radii of curvature and widths and has two essential roles: (1) focusing cells into a linear array at the centre of the straight channel upstream of the pinching region; (2) introducing even spacing between consecutive cells so that cells can reach the imaging region and be analysed one by one^23^. The focusing effect results from a specific ratio between the secondary Dean flow and inertial lift. The latter is a function of the flow rate, cell diameter, and channel’s aspect ratio and radius^42,43^. The longitudinal spacing between consecutive cells depends on the concentration of the cell suspension, the Reynolds number *Re*_*c*_ of the microchannel, and the Reynolds number *Re*_*p*_ of the particle in suspension^24^. The ordered train of cells is then intersected by the side branches where two symmetric fluxes of pure cell-free medium form a pinching flow. Each cell entering this region is subjected to a mechanical deformation resulting from the dynamic interaction with the pinching flow. All measurements were carried out at room temperature 1 hour after cell detachment. The hydrodynamic load was generated by setting a flow rate of 200 μL/min in the cell inlet and 400 μL/min in the pinching flow inlet, respectively.

Cellular viability at the outlet of the microfluidic device was assessed by the exclusion of 0.1% trypan blue dye.

### Cell culture

Healthy cell line PNT2 was purchased from Sigma-Aldrich, while the metastatic prostate cancer cell line DU145 was obtained from ATCC-LGC Standards. Both suppliers ensure authenticated, validated and mycoplasma free cell lines. Cells were cultured in a lab with no history of mycoplasma infection after no longer than 3 months of purchase. DU145 cells were cultured in Corning T-75 flasks (VWR) in Dulbecco’s Modified Eagle Medium/DMEM containing 10% Fetal Bovine Serum and 1% Penicillin-Streptomycin (all Sigma-Aldrich)^43^. PNT2 cells were cultured in Corning T-75 flasks using RPMI-1640 medium (Sigma-Aldrich)^43^. Flasks were kept in a CO_2_ controlled incubator at 37 °C. Samples were prepared for analysis once 70% confluent by removing supernatant media, washing each flask with 5 mL Trypsin-EDTA (Sigma-Aldrich) and incubating with 3 mL Trypsin-EDTA until cells had detached. Trypsin was neutralized adding 7 mL pre-warmed complete RPMI or DMEM^43^. After detachment, cells were spun for five minutes at 1500 RPM and the resulting pellet gently resuspended in 2 mL pre-warmed complete DMEM or RPM. Cells were passaged every third day.

### Imaging

A Nikon Eclipse TE2000-U equipped with a 20x objective was used (Nikon CFI TU Plan Epi ELWD 20×, NA = 0.40, WD = 19.0 mm). A high-speed camera (Fastcam SA-X2, Photron) was employed to image the fast flow-cell interaction. A frame rate of 300,000 fps, a resolution of 256 × 80 pixels and an exposure time of 0.29 μs were set using the proprietary software. The microscope built-in halogen lamp illumination was used.

### Image analysis

An *ad hoc* automated contour tracking algorithm was implemented combining Matlab and C++ and used to quantify the evolution of the cellular deformation over time. The image sequences were analysed by adapting the method reported by Huang *et al*.^44^. Each cell was detected based on its approximate radial symmetry and segmented using a radial active contour model^43^. The cell tracker was initialised once cells first appeared within the field of view. The cell velocity was estimated in each frame and its value used to generate a search region for the cell in the following frame. The cell shape was recorded until exiting the field of view. Short training sequences containing ten randomly selected cells were used to tune the parameters of the algorithm. At each frame the area and perimeter of the cells were determined and the *roundness* calculated as *4πArea/Perimeter*^2^. The code is available upon request.

### AFM Force Spectroscopy

A JPK Nanowizard scanning force microscope (JPK Instruments) positioned on a Zeiss Axiovert inverted microscope (Carl Zeiss AG) was used to perform indentation experiments to gather quantitative measurements of cell stiffness. Well-defined nano-indenters were constructed by adhering 10 μm diameter silica micro-spheres to tipless cantilevers (Arrow TL2, Nanosensors) with a biocompatible two-part epoxy (Araldite). Poly-L-lysine coated coverslips were placed in a custom microscope slide chamber which was subsequently filled with 2 mL of sterile PBS. 20 μL of the cell suspension (~1,000 cells) was pipetted over the immersed coverslip and left motionless for 5 minutes to allow adequate time for all cells to settle into contact with the coverslip. Probe approach speed (1 μm/s) and max indentation force (1 nN) were kept constant for all measurements. Young’s Moduli were determined by fitting the Hertz model to force-indentation curves using a dedicated software package (JPK SPM Data Processing, JPK Instruments).

### Numerical simulation

A fully coupled time dependent fluid-structure interaction finite element model (FEM) of the hydrodynamic stretcher (HS) was created using COMSOL Multiphysics (Comsol Inc.). The model uses an arbitrary Langrangian-Eulerian (ALE) formulation with a deformed mesh to capture the two-way interaction of a deforming cell in the fluid flow. An 800 μm long by 250 μm wide section of the HS, centred on the pinching flow region, was used for the model domain with laminar inflows of 200 μL/min defined at each inlet and a 0 Pa relative pressure boundary condition set at the outlet. Fluid physical properties were set to that of water at room temperature and the cell was defined as a homogeneous linear viscoelastic material.

Values of shear modulus used in the simulation were selected due to the resulting deformation magnitudes approximating the range seen in our experimental observations.

### Statistical analysis

Microsoft Excel, IBM SPSS Statistics 20 (SPSS Inc) and MATLAB 2018b (MathWorks, Inc.) were utilised for the statistical analysis. The functions dscatter and mseb (MathWorks File Exchange) were used to generate the plots in Fig. 2.

Considering that data were not normally distributed the Mann-Whitney U test was performed to assess differences in roundness between DU145 and PNT2 cells analysed using the TR-HS, at three representative points along the profile: initial, maximum and final roundness. The Mann-Whitney U test results are reported indicating the ‘*p-value*’ *and* ‘*Z*’.

All data are available from the authors.

### Cell classification using machine learning

We used machine learning to classify cells as belonging to the two cell lines DU145 or PNT2 based on the cellular feature ‘corrected roundness‘. Specifically, the dependent variable was categorical (DU145 or PNT2) and the independent variables, or predictors, were the different values of the cellular roundness taken at three positions along the temporal profile: initial, final, and maximum. A decision tree classifier was used with cross-validation, specifically the *Simple Tree* implementation in the MATLAB 2016b (MathWorks, Inc.) *Statistics and Machine Learning* Toolbox. Since the single-cell roundness profiles were noisy (due to the outcome of our automatic tracking algorithm), we also trained and validated decision tree classification of groups of five cells known to be from the same class, based on size-corrected roundness features extracted from their average roundness profile.

### Bayesian inference of cell line prevalence

Bayesian inference of prevalence was performed using OpenBUGS (version 3.2.3 rev. 1012). The model used followed that of Speybroeck *et al*.^45^ and assumed that the specificity and specificity reported for the cell classifier in Fig. 3a were correct. Computation of each prevalence posterior density involved running a Markov chain Monte Carlo (MCMC) sampling algorithm. Specifically, a single chain of 20,000 samples was generated.

## Supplementary information

TRHS Supplementary Information

2a

2bc

3a

3b

3c
