# Supplementary material for: High-Throughput, Time-Resolved Mechanical Phenotyping of Prostate Cancer Cells
Source: Sci Rep. 2019 Apr 5;9:5742. doi: 10.1038/s41598-019-42008-0 (PMC6450875; doi:10.1038/s41598-019-42008-0)
Supplement: Supplementary file 1 — TRHS Supplementary Information [file 41598_2019_42008_MOESM1_ESM.pdf]

Supplementary Information

High-Throughput, Time-Resolved Mechanical Phenotyping of Prostate Cancer Cells

Yuri Belotti[1*], Serenella Tolomeo[3**], Michael J Conneely[2**], Tianjun Huang[1**], Stephen J. McKenna[1], Ghulam Nabi[3], David McGloin[1,4*].

[1]University of Dundee, SUPA, School of Science and Engineering, Dundee, Scotland, UK

[2]University of Dundee, School of Life Sciences, Dundee, Scotland, UK

[3]University of Dundee, School of Medicine, Dundee, Scotland, UK

[4]University of Technology Sydney, School of Electrical and Data Engineering, Sydney, Australia

[*]Corresponding author: david.mcgloin@uts.edu.au

[**]These authors contributed equally to this work

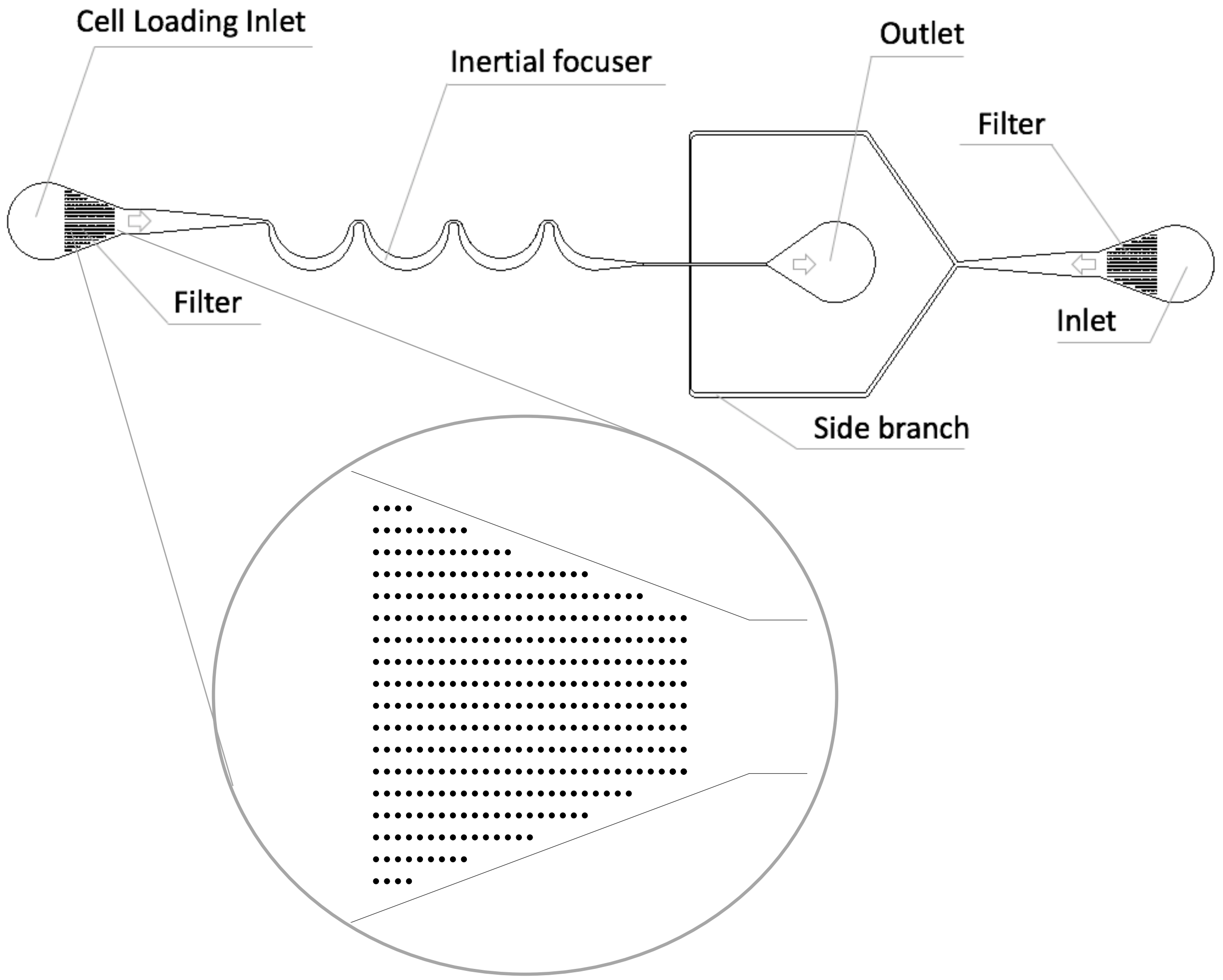

Parameter	Small bend	Big bend	Unit	Description
**W**	98	419	µm	*Width*
**H**	30	30	µm	*Height*
**R**	168	1330	µm	*Radius of curvature*
D_h	45.94	55.99	µm	*Hydraulic diameter*
R_e	208.33	59.39		*Reynolds number*
D_e	77.03	8.62		*Dean number*

Hydrodynamic parameters of the small and big bends that compose the inertial focuser.

Parameter	Side branches	Main channel	Unit	Description
**W**	60	60	µm	*Width*
**H**	30	30	µm	*Height*
D_h	40	40	µm	*Hydraulic diameter*
v_{mean}	2.84	2.84	m/s	*Flow speed*
R_e	113.6	113.6		*Reynolds number*

Hydrodynamic parameters of the main and side branches at the pinching region.

Figure S1: CAD design of the microfluidic chip. Cell suspension and ‘pinching medium’ are loaded through the inlet on the left-hand side, and right-hand side, respectively. At each inlet, a filter prevents big particulate and unwanted objects from entering the microchannels. The flow rate of the two fluxes is controlled separately using independent modules of the syringe pump.

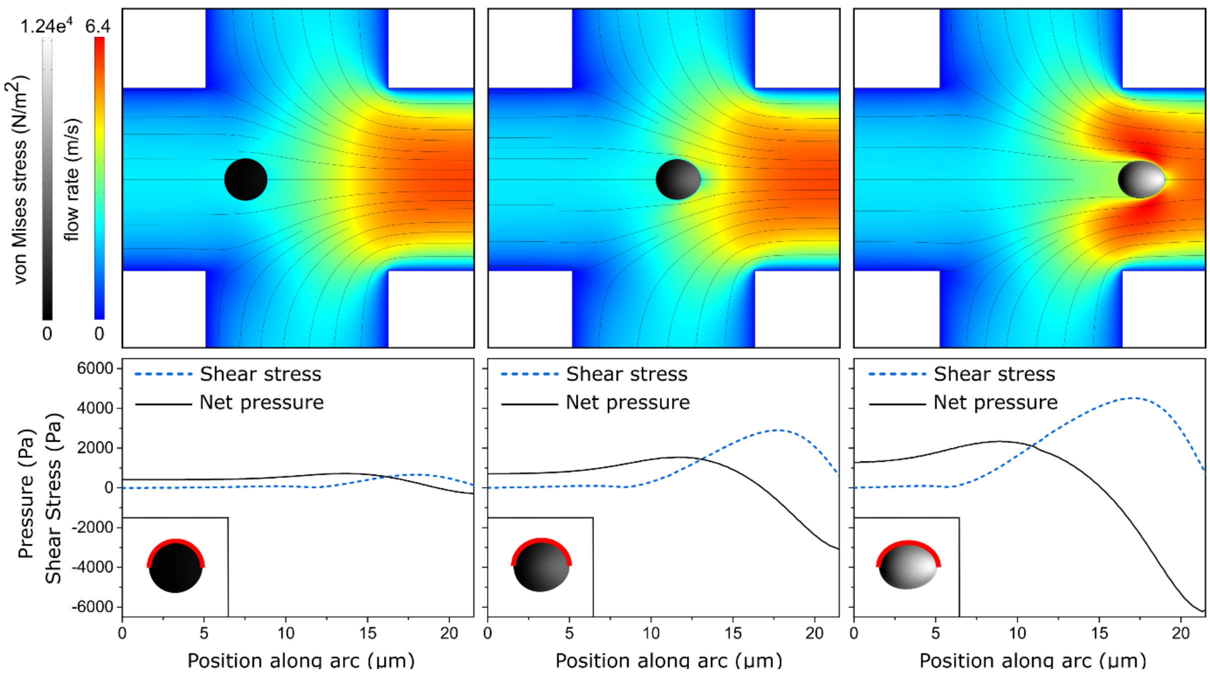

Figure S2: Time evolution of stress profiles across a cell passing through the pinched flow region in the hydrodynamic stretcher (HS). Stress profiles across a cell passing through the pinched flow region of the HS. Upper panels show a homogeneous linear viscoelastic cell with a shear modulus of 45 kPa at three time-points from a time-dependent fully coupled fluid-structure interaction computational model. Fluid flow rates in the channel and von Mises stress in the cell are depicted by the corresponding colour scales. Lower panels show corresponding line plots across the top perimeter of the cell (profiles indicated by red outlines and are identical for lower half of the cell) indicating the rapid change in applied net pressure and shear stress across the cell as it passes through the pinched flow region.

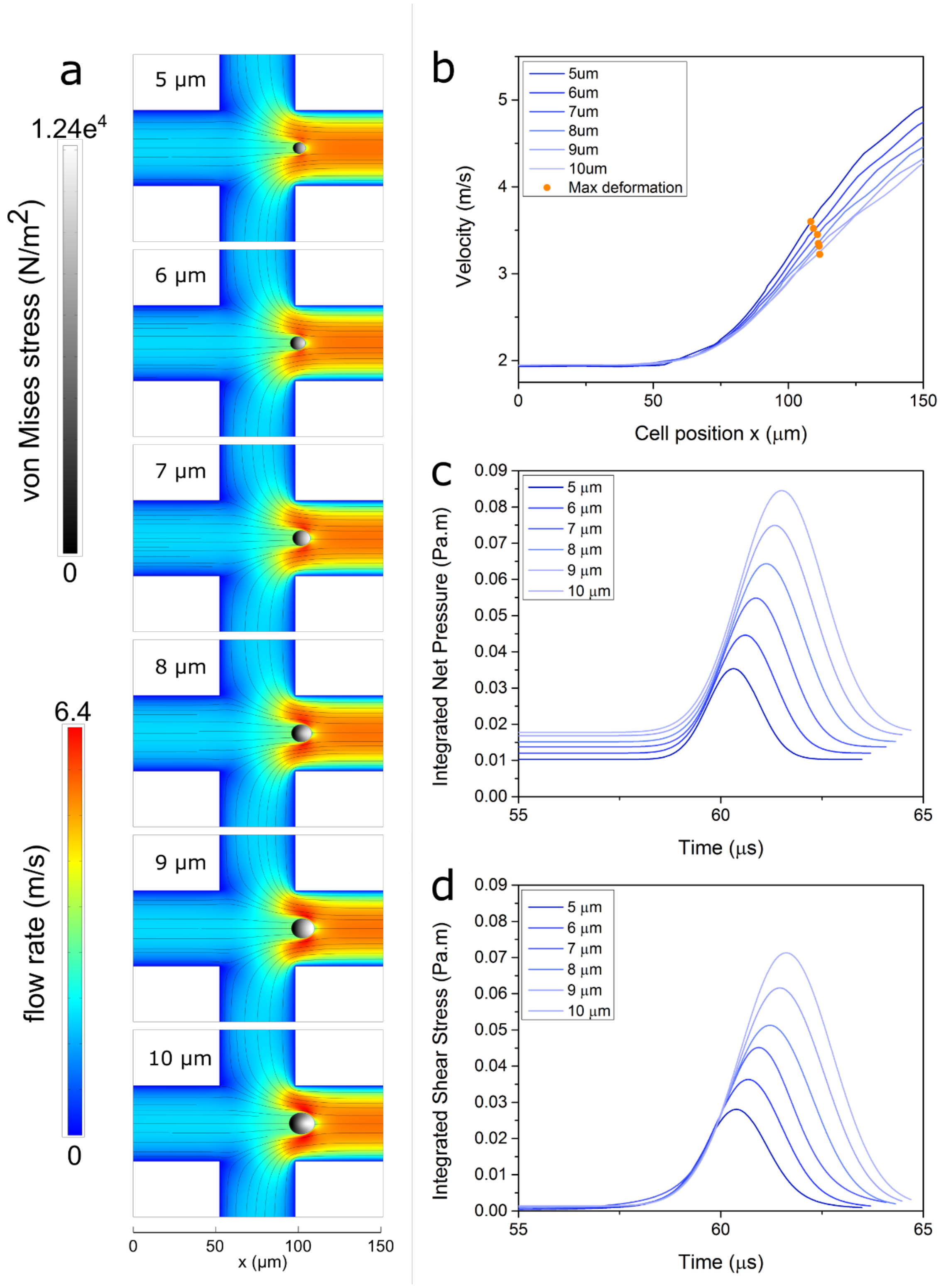

Figure S3: Relationship between cell size, cell speed and hydrodynamic forces. (a) A series of images depicting the position of maximum deformation for cells of different radii (5 – 10 μm) passing through pinched flow region. Respective colour scales indicate fluid flow rates and stress magnitudes. (b) Cell velocity plot where cell position is calculated as centroid of deformed cell geometry. Cells enter pinched flow region under a steady state flow and accelerate towards the outlet. Respective positions of maximum deformation are indicated by circle markers. Larger cells accelerate slower and experience their maximum deformation at a small displacement downstream when compared to smaller cells. Furthermore, larger cells undergo their maximum deformation at a lower velocity. (c) Plot of net hydrodynamic pressure integrated over cell perimeters. (d) Plot of shear stress integrated over cell perimeters. Note that peak values of net pressure and shear stress for larger cells are delayed if compared with smaller cells, due to relative velocity differences.

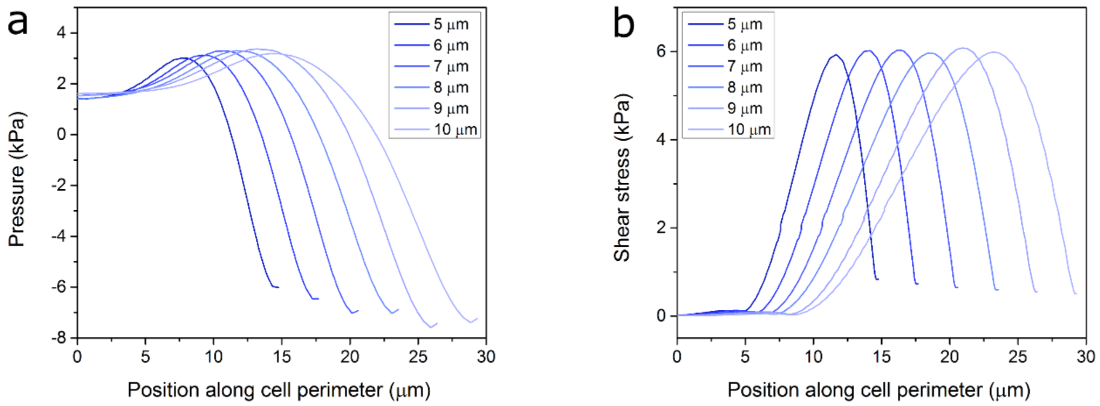

Figure S4: Relationship between cell size and hydrodynamic forces. Hydrodynamic pressure (a) and shear stress (b) profiles calculated in a similar fashion to those featured in Supplementary Figure 2. Line plots for cells of varying radii are plotted at the time of maximum deformation. It is noteworthy that cells of different radii experience similar maximal stress however, as shown in Supplementary Figure 3c and Supplementary Figure 3d, when integrated over an entire cell perimeter, larger cells experience a greater total amount of force when passing through the pinched flow region.

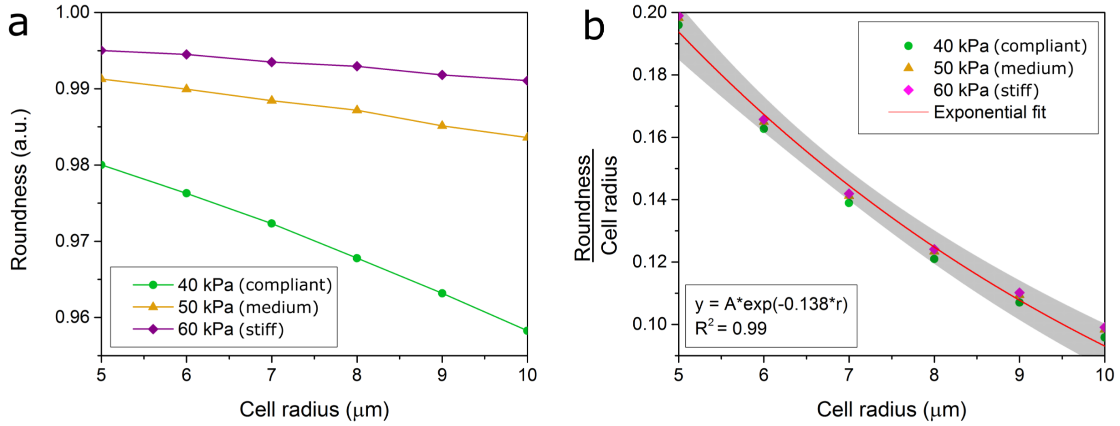

Figure S5: Cell size effect on maximum observed deformation. (a) Plot of maximum deformation (roundness of 11 would indicate no deformation) for cells of various sizes and shear moduli. (b) Roundness values were normalised for cell radii and an exponential fit was made to the data. When normalised, each cell shear moduli group displayed a similar trend. Shaded area represents the 95% confidence bands of the exponential fit.

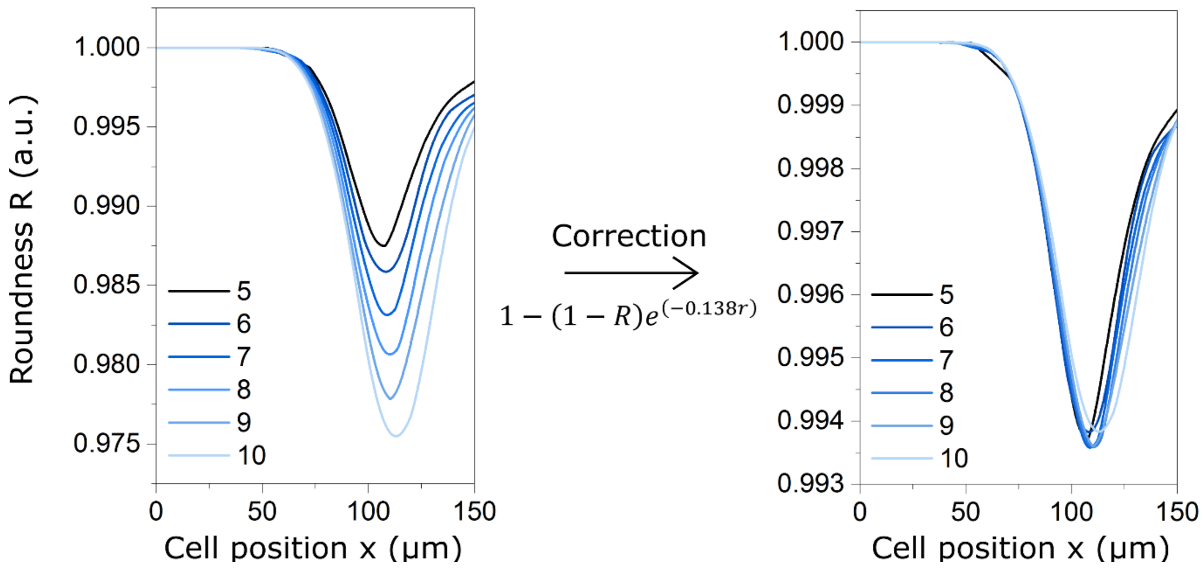

Figure S6: Dimensionless correction factor for cell size. Left panel shows the time resolved roundness profiles for cells with a shear modulus of 45 kPa and radii ranging from 5 - 10 µm in the FEM model. Variability in the observed roundness due to effect of cell size is reduced by a factor greater than 4 when a dimensionless correction factor based on the exponential fit in Figure S5 is applied, decreasing the absolute variability to less than 10% while maintaining the overall time dependent evolution of the cell roundness profiles. R = cell roundness. r = initial cell radius.

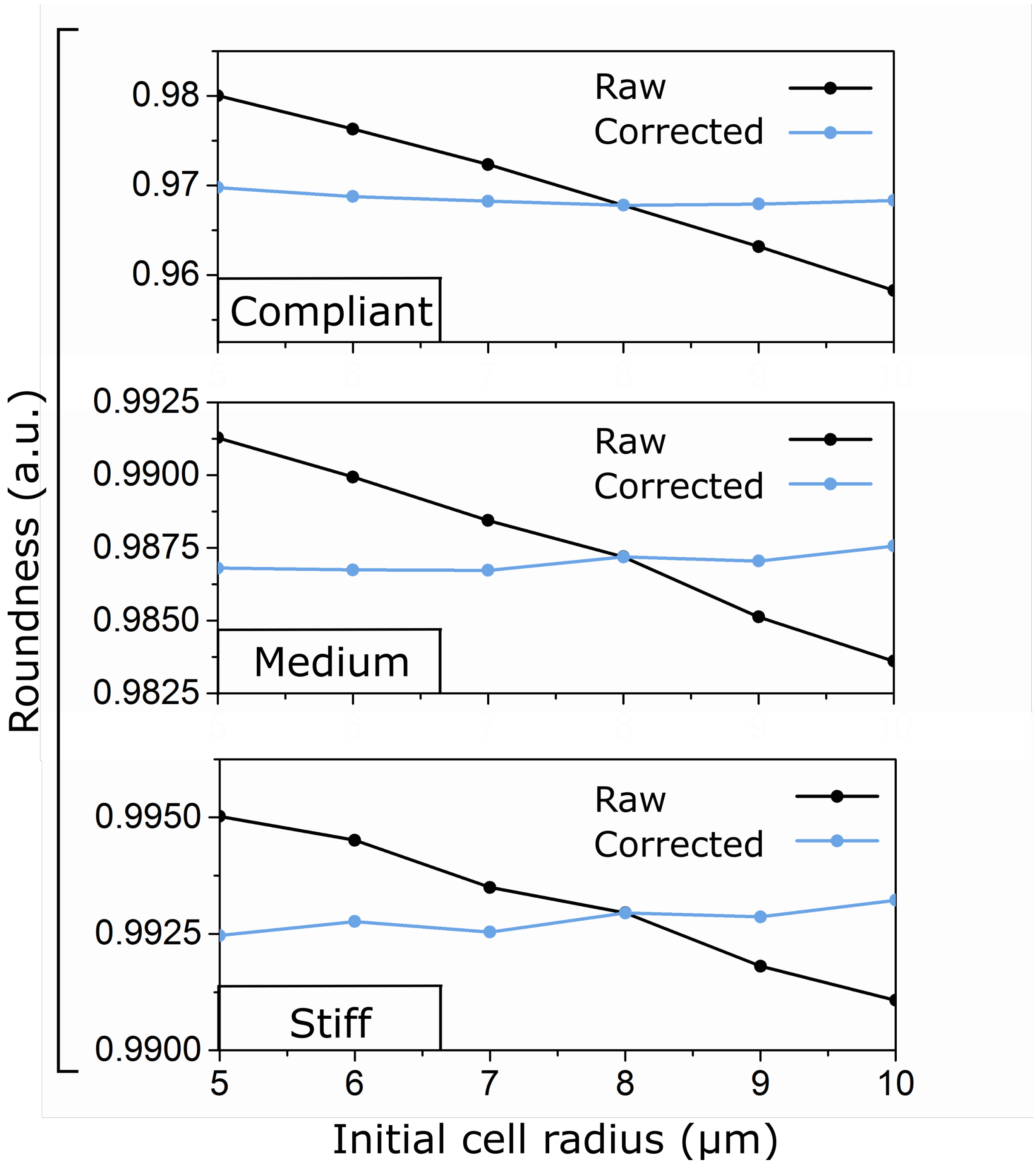

Figure S7: Robustness test of cell size correction for cells of different elasticities. The correction factor presented in Figure S6 was applied to cells of differing stiffness. Shear moduli of 40 kPa, 50 kPa, and 60 kPa were used to simulate “compliant”, “medium” and “stiff” cells respectively (this range of deformability reflects that observed in the TR-HS experiments). Plots show the roundness values at peak deformation for each cell size and elasticity. The correction factor was applied and the resulting corrected roundness values were normalised back to the 8 μm raw value to allow a clearer illustration of the magnitude of correction.

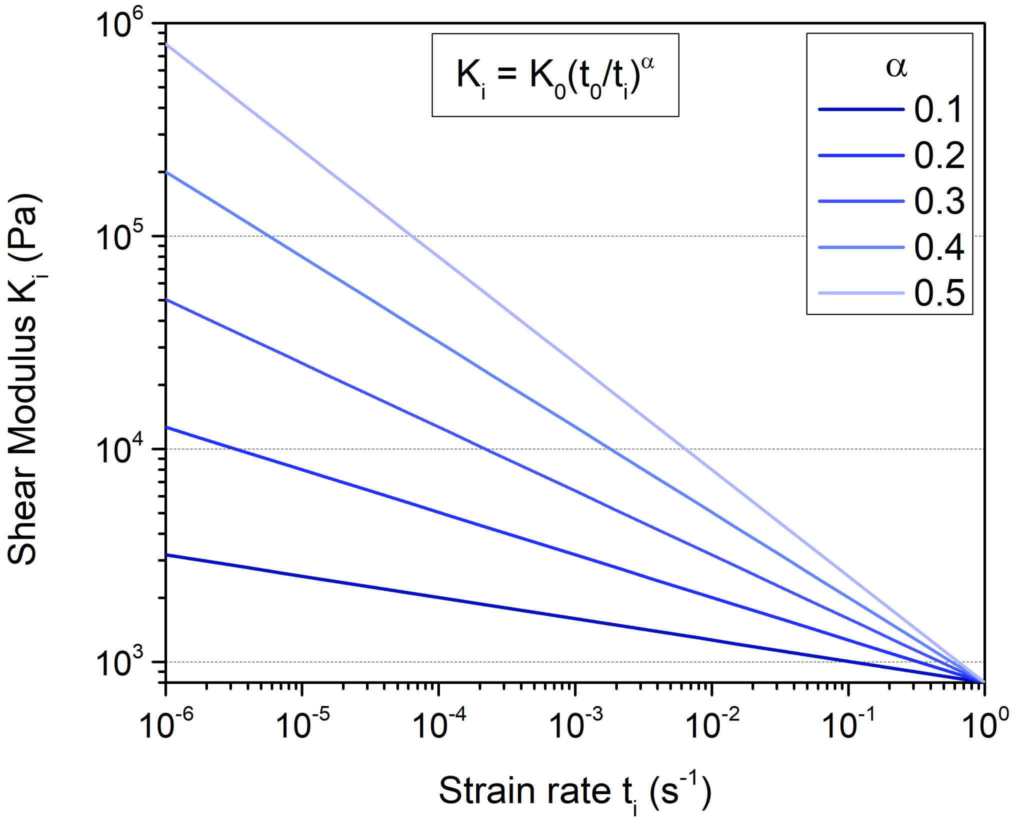

Figure S8: Power-low description of cellular elastic response. Plot of power-law extrapolation from AFM estimated elastic modulus of 799 Pa (K_0) at a strain rate of 1 s^{-1}(t_0). Extrapolating to strain rates extending to1 μs^{-1}(as experienced by cells in the case of the TR-HS), the relation points to elastic moduli ranging from ~3-800 kPa which is fitting to the results obtained from the FEM simulations.

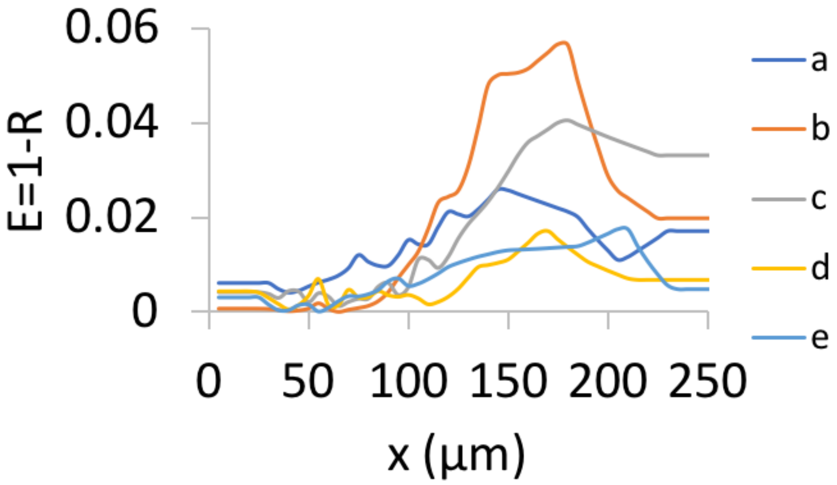

Figure S9: Single-cell spatial profiles of the deviation from the perfect roundness E=1-R extracted using the automated tracking algorithm of 5 consecutive cells (a-e) passing through the microchannel.

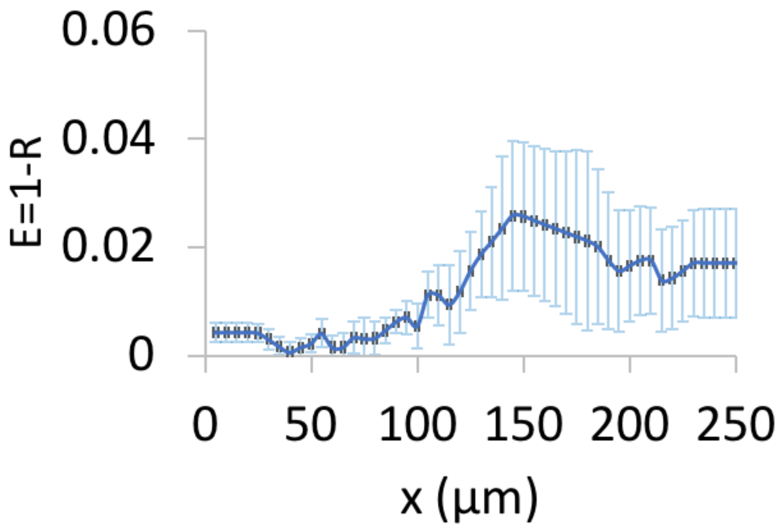

Figure S10: Median of the single-cell spatial profiles shown in Figure S9. Error bars represent 95% confidence interval.

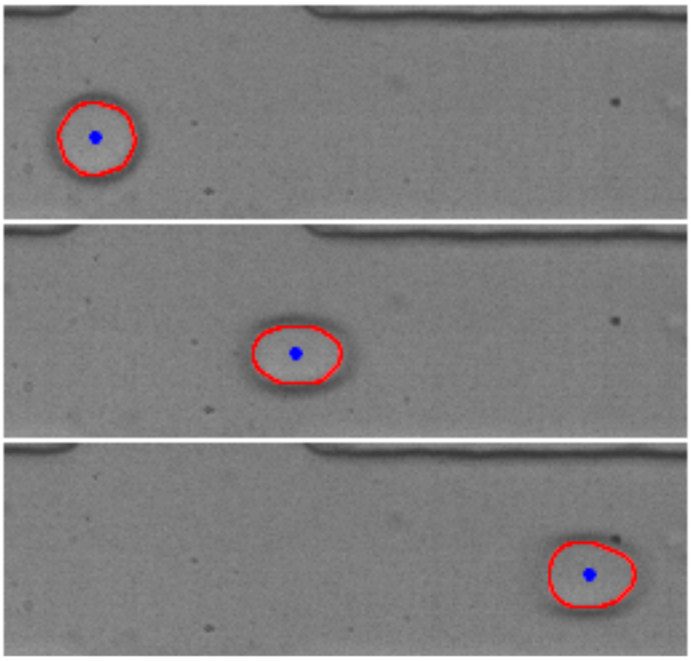

Figure S11: Representative pictures showing the output of the automated tracking algorithm. The blue dot represents the centroid of the cell, the red line represents the cell outline.
